# Recommendations for the return of spectators to sport stadiums: A South African Sports Medicine Association (SASMA) position statement – Part 4

**DOI:** 10.17159/2078-516X/2021/v33i1a12558

**Published:** 2021-01-15

**Authors:** L Pillay, J Patricios, DC Janse van Rensburg, R Saggers, D Ramagole, P Viviers, C Thompson, S Hendricks

**Affiliations:** 1 Section Sports Medicine & Sport, Exercise Medicine and Lifestyle Institute (SEMLI), Faculty of Health Sciences, University of Pretoria, Pretoria, South Africa; 2 Wits Sport and Health (WiSH), Faculty of Health Sciences, University of the Witwatersrand, Johannesburg, South Africa; 3 Premier Soccer League COVID-19 Chief Medical Officer, PSL, South Africa; 4 Chief Medical Officer, Gauteng Lions Cricket, South Africa; 5 Medical Board Member, World Netball, Manchester, UK; 6 Campus Health Service, Stellenbosch University, South Africa; 7 Institute of Sport and Exercise Medicine, Division of Orthopaedic Surgery, Department of Surgical Sciences, Faculty of Medicine and Health Sciences, Stellenbosch University, South Africa; 8 FIFA Medical Centre of Excellence, South Africa; 9 Department of Paediatrics and Child Health, Charlotte Maxeke Johannesburg Academic Hospital, Johannesburg, South Africa; 10 Division of Physiological Sciences, Department of Human Biology, Faculty of Health Sciences, University of Cape Town, South Africa; 11 Health through Physical Activity, Lifestyle and Sport (HPALS) Research Centre, Department of Human Biology, Faculty of Health Sciences, University of Cape Town. South Africa; 12 Carnegie Applied Rugby Research (CARR) centre, Institute for Sport Physical Activity and Leisure, Leeds Beckett University, Leeds, England

**Keywords:** COVID-19, risk-mitigating, events, spectators, sports

## Abstract

All sports were discontinued in 2020 with the arrival of COVID-19. Since then most have been reinstated, albeit without spectators. However, several countries have put together a number of different risk-mitigating strategies to allow spectators back into stadiums. This position statement gives an outline of the minimum requirements that should be considered upon the return of spectators at live sporting events.

Several sporting codes have returned to competition around the world. Recently, the Tokyo Olympics and Paralympics, postponed from 2020, concluded safely, albeit with no spectators and the rigorous daily testing of participants.^[1]^

In South Africa, all sporting federations were given the green light to resume professional sport on 20 August 2020 by the Ministry of Sports, Art and Culture.^[2]^ This resumption was based on specific conditions, such as COVID-19 PCR tests, continuous education, daily screening, and no spectators allowed. Early this year, the South African Sports Confederation and Olympic Committee (SASCOC), together with the Event and Safety Council (ESC), was mandated by the Department of Sports, Culture and Recreation (DSACR) to develop a document to explain what processes should be put in place for the safe return of spectators.^[3]^

The first country to host spectators after COVID-19 was declared a pandemic was Qatar. This was in December 2020 at the Amir Cup Final.^[4]^ A total of twenty thousand spectators were allowed to enter (50% of the stadium capacity) subject to a negative antigen test not older than 72 hours. Social distancing, temperature screening and mask-wearing (non-pharmaceutical interventions or NPIs) were mandatory before entry. The results of the antigen test and monitoring were captured using a geo-locating cell phone app and online booking system. While this was a costly exercise for both the organisers and spectators (the spectator had to pay for any testing that was required), as far as the researchers were aware, the Final was a success in terms of managing the risk of COVID-19. The researchers did not have access to the post-match track and trace app statistics for spectators.

The Euro 2020 UEFA competition (held in 11 different European countries from 11 June 2021 to 11 July 2021) also allowed spectators.^[5]^ Similar to the Amir Cup Final, the spectators of the Euro 2020 UEFA competition had to manage the COVID-19 risk using NPIs and a negative COVID-19 test. International experience shows that in the time leading to the Euro 2020 UEFA competition, COVID-19 vaccines were also available, which added another risk mitigating layer to reduce the spread of the COVID-19 virus.

## Recommendations for spectators at stadiums for professional sport

Considering the above, the following is recommended to allow spectators to return safely to stadiums in South Africa. Any competition wanting to return spectators should consult the relevant stakeholders and would be required to comply with the DSACR and Department of Health regulated requirements. This would include the National Federations together with the ESC:

### Stadium protocols

1. There should be a stepwise approach for allowing fans into stadiums; for example, initially at 10% of venue capacity, then 25%, then 50% (within keeping to the Disaster Management Act regulation regarding indoor and outdoor maximum capacities). It will also allow for appropriate social distancing to occur (at least one empty seat between spectators). Seats must be allocated, and spectators must utilise these allocated seats as this is vital for contact tracing. Stadiums and spectators will be able to familiarise themselves with the logistics and processes required. After every event, there should be a post-event dissection of the pearls and pitfalls and improvements accordingly prior to moving to the next level of spectator allowance.
2. Stadium protocols are already in place regarding the sanitisation of the stadium, ablution facilities, and easily available hand sanitisers/handwashing stations. However, these may need to be increased. Staggered ingress and egress of stadiums are required, ensuring there are no mass crowd gatherings, and social and physical distancing (at least 1.5 metres apart).
3. COVID-19-related educational posters around the stadium must remind spectators to keep a social distance of at least 1.5 m, wearing masks properly and sanitising of their hands.
4. Staff working at the event must be fully vaccinated.
5. Should there be an indoor event, cross ventilation ^[6]^ is vital and high efficiency particle air (HEPA) purifiers should be considered.^[7]^

### Mandatory spectator conditions

6. Mandatory mask-wearing covering the nose and mouth at all times.
7. Proof of full vaccination (this means 14 days after a Johnson and Johnson vaccination or 2 weeks after the second Pfizer vaccination) ^[8]^ needs to be provided – this should be confirmed when purchasing the ticket and at entry into the stadium; proof of vaccination must be cross-checked against the national electronic vaccination data system (EVDS).
8. Tickets cannot be transferred.
9. Spectators must also realise that even though all protocols are observed, the risk of contracting COVID-19 cannot be mitigated 100%. This should be covered in a disclaimer when purchasing the ticket.

### Screening and surveillance

10. A mobile COVID-19 screening app with questions to screen symptoms and exposure in the last 14 days.
11. The World Health Organization (WHO) Mass Gathering COVID-19 Risk Assessment Tool - Sports Events should be utilised by the event planning team. ^[9]^
12. There must be a system in place for spectators (who, within five days of the event, find out they have tested positive for COVID-19) to contact and advise planners so that contact tracing can be undertaken.
13. Stadiums must also have the ability to do rapid antigen tests onsite, or be able to transport an attendee (spectator or staff) to the nearest antigen test site, should an attendee screen positive for any COVID-19 symptoms.
14. Constant countrywide surveillance of COVID-19 transmission patterns must be a major dictator of whether or not spectators may attend events.

## Recommendations for spectators in amateur sport – club and school

1. The usual NPI protocols must continue.
2. All spectators must be screened (via symptom questionnaire and temperature) prior to entry into the stadium.
3. There should be a maximum capacity of 50% of spectators or as dictated by the gazetted regulations by the Minister of Sports, Art and Culture.
4. Where an event is indoors, cross ventilation is vital and high efficiency particulate air (HEPA) purifiers should be considered.
5. Anyone screened at the event who poses a risk of recent exposure to COVID-19 or presents with symptoms must not be allowed entry.
6. Constant countrywide surveillance of COVID-19 transmission patterns must be a major dictator of whether or not spectators may attend events.

## Conclusion

This pandemic will likely last for many more months or even years. Vaccination is most important and should be encouraged as this will assist in returning to the pre-pandemic status quo as per the government’s new vaccination campaign on ’Return to Play ‘^[10]^. With evolving evidence, changes should be implemented regularly. New virus mutations will occur. However, we should have a malleable plan to allow spectators to socialise safely with all risk mitigating approaches and protocols in place.
